# Fibrosis versus plasticity: a tensegrity-based framework for acupuncture as an exploratory modulator of tissue recovery

**DOI:** 10.3389/fphys.2026.1779725

**Published:** 2026-05-29

**Authors:** Alejandro Carballo

**Affiliations:** 1 Nässjö Läkarhus, Region Jönköping County, Jönköping, Sweden; 2 Futurum – Academy for Health and Care, Region Jönköping County, Jönköping, Sweden

**Keywords:** acupuncture, fascia, fibrosis, mechanotransduction, plasticity, recovery, tensegrity, viscoelasticity

## Abstract

**Background:**

Fibrosis represents a progressive loss of tissue adaptability characterized by increased extracellular matrix (ECM) stiffness, impaired mechanotransduction, and reduced capacity for tonus and form restoration. Although fibrosis is commonly investigated at molecular or organ-specific levels, its system-wide biomechanical implications remain insufficiently explored. Tensegrity theory provides an architectural framework in which connective tissue functions as a pre-stressed, load-redistributing network.

**Objective:**

To propose a mechanistic model in which acupuncture is conceptualized as a localized mechanical perturbation within a tensegrity-regulated connective tissue system, with potential to modulate the fibrosis–plasticity continuum and support recovery of impaired tissue adaptability.

**Methods:**

A conceptual framework integrating connective tissue biomechanics, fibroblast mechanosensing, and viscoelastic remodeling is developed. Neuroendocrine mechanisms are incorporated as secondary modulators influencing response velocity and variability rather than primary causal drivers. Measurable biomechanical proxies are defined to enable empirical testing.

**Conclusion:**

A tensegrity-based interpretation allows acupuncture to be studied as an architectural intervention targeting impaired plasticity rather than as a purely neuro-reflexive therapy. This framework enables hypothesis-driven exploration of recovery trajectories in fibrotic, age-related, and chronic functional conditions.

## 1 Introduction

Fibrosis is increasingly recognized as a shared downstream phenotype across a wide range of chronic conditions, including cardiovascular disease, neurodegeneration, metabolic disorders, and musculoskeletal aging (Rockey et al., 2015; Henderson et al., 2020). Despite heterogeneous etiologies, fibrotic tissues exhibit convergent biomechanical characteristics: increased stiffness, altered strain transmission, and diminished reversibility (Wells, 2013; Hinz, 2015). Despite extensive molecular characterization, current models of fibrosis remain limited in their ability to predict functional recovery, suggesting that critical system-level determinants are not captured by existing frameworks. We argue that this limitation reflects the absence of an architectural perspective linking molecular events to tissue-level mechanics and whole-body force distribution.

Recovery from chronic disease or injury depends not only on molecular resolution but on the capacity of tissues to restore baseline tonus, redistribute mechanical strain, and re-establish adaptive deformation patterns (Liu et al., 2010; Hinz and Lagares, 2020). This capacity is herein referred to as plasticity. We propose that fibrosis and plasticity should be understood as architectural states within a continuous biomechanical system rather than as discrete pathological entities.

### 1.1 Tensegrity as an organizing principle

A key insight from biomechanics is that living tissues function as tensegrity systems—structures where global stability emerges from the interaction between continuous tensile elements (ECM fibers, fascial networks) and discontinuous compression elements (cells, bone, cartilage) (Pugh, 1976; Motro, 2003; Skelton and de Oliveira, 2009). Originally developed in architecture and engineering, tensegrity principles have been successfully applied to cytoskeletal organization, cellular mechanics, and tissue architecture (Ingber, 2003a; Ingber, 2003b; Ingber, 2006).

In tensegrity systems, mechanical forces distribute globally through prestressed networks, enabling non-local effects from localized perturbations (Scarr, 2010; Turvey and Fonseca, 2014). Recent advances in tensegrity modeling have clarified:

- Stability conditions considering both local and global buckling behavior (Ma and Chen, 2025)
- Seven decades of theoretical and experimental development documenting the robustness of tensegrity principles across scales (Micheletti and Podio-Guidugli, 2022)
- Mechanism creation through cellular morphogenesis, demonstrating how tensegrity governs tissue organization during development and remodeling (Aloui and Rhode-Barbarigos, 2020)

This architectural perspective provides a mechanistic foundation for understanding how localized mechanical interventions might produce system-wide functional effects without requiring continuous anatomical conduits or purely neural mediation. In this framework, tensegrity is not employed as a descriptive metaphor but as a constraint-based model that defines which mechanical perturbations can propagate across scales and which remain locally dissipated. This distinction is critical: if mechanically induced perturbations fail to propagate beyond local tissue domains, the tensegrity-based interpretation would be invalid.

### 1.2 Acupuncture as a mechanical perturbation

Within this context, acupuncture may be explored as a mechanical intervention capable of influencing tissue-level plasticity through tensegrity-mediated mechanisms. Rather than invoking metaphysical constructs, this framework positions acupuncture as a reproducible localized mechanical stimulus whose effects can be investigated through established principles of mechanobiology and systems physiology (Langevin et al., 2001a; Langevin et al., 2005; Langevin et al., 2006).

This manuscript develops a conceptual framework integrating connective tissue biomechanics, fibroblast mechanosensing, and viscoelastic remodeling to generate testable hypotheses about acupuncture’s potential role in modulating the fibrosis–plasticity continuum.

## 2 Fibrosis as loss of architectural plasticity

### 2.1 Beyond molecular models: mechanical memory and tissue stiffness

Traditional models of fibrosis emphasize fibroblast activation, cytokine signaling (particularly TGF-β), and excessive ECM deposition (Wynn and Ramalingam, 2012; Rockey et al., 2015; Henderson et al., 2020). While these molecular processes are essential, they do not fully explain several clinical observations:

- Persistent functional rigidity following biochemical normalization (Kass et al., 2004; Borlaug and Paulus, 2011)
- Delayed or incomplete clinical recovery despite anti-inflammatory treatment (Wells et al., 2003; King et al., 2014)
- Discrepancies between imaging findings and symptom burden (Provenzano and Vanderby, 2006; Scarcelli et al., 2015)
- Fibrosis also involves mechanical memory, whereby tissues retain prior stress states through cytoskeletal reorganization and altered ECM architecture (Kjaer et al., 2009; Balestrini et al., 2012).

Fibroblasts cultured on stiff substrates maintain activated phenotypes even when transferred to soft environments, demonstrating cellular-level mechanical memory (Engler et al., 2006; Yang et al., 2014). At the tissue level, this mechanical memory contributes to impaired adaptability even when inflammatory activity subsides (Wynn, 2008; Kis et al., 2011).

### 2.2 Definition of plasticity

Plasticity is defined here as the tissue-level capacity to restore baseline tonus and form following mechanical perturbation, enabling adaptive redistribution of strain over time. This definition encompasses:

- Viscoelastic reversibility: Return toward baseline state after deformation (Findley et al., 1989; Lakes, 2009)
- Distributed strain transmission: Spread of mechanical loads across extended tissue networks (Gao et al., 2011; Screen et al., 2015)
- Adaptive remodeling: Structural reorganization supporting functional demands without pathological rigidification (Lutolf and Hubbell, 2005; Humphrey et al., 2014)

Conceptual hypothesis: Loss of plasticity may precede overt fibrosis and constitute a pre-fibrotic architectural state with clinical relevance. This hypothesis predicts that interventions targeting mechanical properties during early plasticity loss could prevent progression to structural fibrosis. Importantly, plasticity is not equivalent to elasticity; tissues may recover position without restoring distributed strain behavior, resulting in functionally rigid yet superficially normal mechanical states.

### 2.3 The fibrosis–plasticity continuum

**Figure 1:**
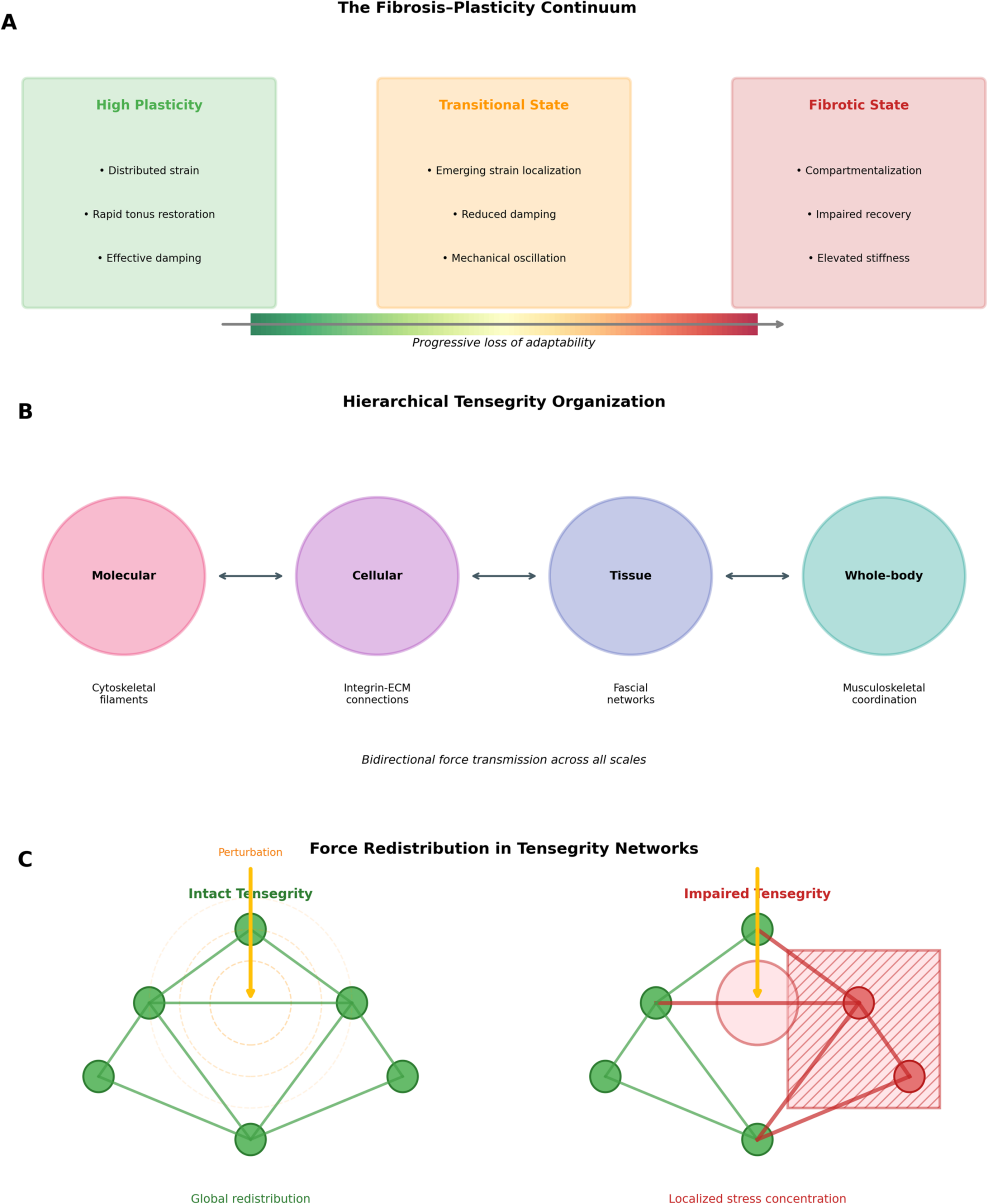
The Fibrosis–plasticity continuum and tensegrity architecture. **(A)** *The Fibrosis–Plasticity Continuum.* Schematic representation of tissue states along a mechanical continuum. Left (High Plasticity): Tissue exhibits distributed strain transmission (arrows spread widely), rapid tonus restoration after perturbation, and effective viscoelastic damping. Center (Transitional State): Emerging strain localization (arrows concentrate), reduced damping capacity, increased mechanical oscillation. Right (Fibrotic State): Mechanical compartmentalization (arrows blocked), impaired recovery, elevated baseline stiffness. Gradient bar indicates progressive loss of adaptability. **(B)** *Hierarchical Tensegrity Organization.* Four nested levels of tensegrity architecture in biological systems. Molecular level: Cytoskeletal filaments (actin in red, microtubules in green, intermediate filaments in blue) providing intracellular prestress through continuous tension with discontinuous compression-bearing elements. Cellular level: Integrin-mediated connections (purple) linking intracellular cytoskeleton to extracellular matrix, transmitting forces bidirectionally across cell membrane. Tissue level: Fascial networks forming continuous tensile pathways (yellow sheets) with embedded fibroblasts, connecting muscle compartments and transmitting strain across anatomical regions. Whole-body level: Integrated musculoskeletal system with bones (compression elements) and myofascial chains (tensile elements) distributing gravitational and movement-induced loads. Bidirectional arrows indicate force transmission across all scales. **(C)** *Force Redistribution in Tensegrity Networks.* Comparison of mechanical perturbation effects in intact versus impaired tensegrity. Top (Intact Tensegrity): Local perturbation (acupuncture needle insertion, black arrow) produces global redistribution of tension throughout the prestressed network. Colored lines indicate stress redistribution reaching distant regions, with force magnitude decreasing gradually with distance (color gradient from red to blue). Bottom (Impaired Tensegrity): Same local perturbation in fibrotic tissue produces localized stress concentration. Force transmission is blocked by stiffened regions (hatched areas), creating mechanical compartments. Stress remains high near perturbation site (red) with minimal distant effects (no blue regions).

Conceptual framework: Rather than binary states, tissues occupy positions along a continuum:

- High plasticity: Preserved tonus restoration, adaptive strain redistribution, effective viscoelastic damping
- Transitional state: Reduced damping, increased mechanical oscillation, emerging strain localization
- Fibrotic state: Elevated stiffness, impaired tonus recovery, mechanical compartmentalization (**Figure 1**)

This continuum model explains why functional improvement may precede detectable molecular or imaging changes—tissues can shift toward greater plasticity through altered mechanical organization without requiring complete structural reversal (Discher et al., 2005; Tschumperlin et al., 2013). This framework predicts that early interventions should first modify viscoelastic damping properties before detectable changes in bulk stiffness occur, providing a potential temporal hierarchy for measurable recovery.

## 3 Tensegrity principles applied to connective tissue architecture

### 3.1 Hierarchical tensegrity organization

Tensegrity describes systems stabilized by continuous tension and discontinuous compression (Motro, 2003; Skelton and de Oliveira, 2009; Ma and Chen, 2025). Applied to biology, it conceptualizes the human body as a hierarchically nested mechanical network encompassing:

- Molecular level: Cytoskeletal filaments (actin, intermediate filaments, microtubules) providing intracellular prestress (Ingber, 2006; Wang et al., 2009)
- Cellular level: Cell-ECM interactions through integrin-mediated focal adhesions transmitting forces bidirectionally (Geiger et al., 2009; Hynes, 2009)
- Tissue level: Fascial networks, myofascial chains, and organ capsules forming continuous tensile pathways (Schleip et al., 2006; Stecco et al., 2008)
- Whole-body level: Integrated musculoskeletal coordination distributing gravitational and movement-induced loads (Chen and Ingber, 1999; Levin, 2002)

### 3.2 Prestress and force redistribution

A critical feature of tensegrity systems is baseline mechanical tension (prestress) that stabilizes architecture and enables immediate force redistribution (Sultan et al., 2004; Vera et al., 2005). In biological tissues:

- Prestress is actively maintained through cellular contractility (myofibroblasts, smooth muscle) and ECM organization (Tomasek et al., 2002; Hinz, 2012)
- Local perturbations propagate globally through the prestressed network, producing non-local mechanical effects (Scarr, 2010; Turvey and Fonseca, 2014)
- Loss of prestress or uneven distribution increases susceptibility to mechanical failure (Stamenović et al., 1996; Liedl et al., 2010)

Conceptual hypothesis: Fibrotic progression represents breakdown of effective tensegrity behavior, resulting in uneven prestress distribution, shortened strain-propagation lengths, and reduced capacity for global force redistribution.

### 3.3 Fascia as the primary tensegrity network

Fascia forms a body-wide continuous network of connective tissue (Stecco et al., 2011; Benias et al., 2018). Recent research has demonstrated:

- Fascial layers are mechanically interconnected across anatomical regions (Stecco et al., 2009; Adstrum et al., 2017)
- Fascia exhibits active contractile properties mediated by myofibroblasts (Hinz et al., 2012; Schleip et al., 2019)
- Fascial innervation includes mechanoreceptors capable of detecting tissue deformation (Yahia et al., 1992; Tesarz et al., 2011)

Relevance to framework: Fascia represents the primary substrate through which tensegrity-based force redistribution occurs at the whole-body level.

## 4 Acupuncture as a localized mechanical perturbation

### 4.1 Reframing the intervention

Conceptual framework: Within the tensegrity model, acupuncture is conceptualized as a localized mechanical perturbation applied to a pre-stressed connective tissue network. Needle insertion and manipulation induce:

- Focal fascial deformation and tissue displacement (Langevin et al., 2001a; Langevin et al., 2006)
- Altered local strain vectors and stiffness gradients (Chaudhry et al., 2008; Roman et al., 2013)
- Transient modification of prestress distribution (Cao et al., 2013; Cao et al., 2015)

Acupuncture needles mechanically engage subcutaneous connective tissue:

- Needle rotation produces connective tissue winding and pulling (Langevin et al., 2004; Langevin et al., 2011a)
- Tissue displacement extends several millimeters from needle insertion point (Langevin et al., 2002; Langevin et al., 2013)
- Mechanical coupling between needle and tissue can be quantified via pull-out force measurements (Dorsher and Fleckenstein, 2008; Huang et al., 2012)

Conceptual hypothesis: These perturbations propagate along preferential mechanical pathways determined by tissue architecture and stiffness distribution rather than by discrete anatomical conduits. These perturbations likely operate at strain magnitudes below macroscopic deformation thresholds yet sufficient to alter cytoskeletal tension and mechanotransductive signaling in resident fibroblasts.

### 4.2 Distinguishing acupuncture from other mechanical stimuli

How does acupuncture differ mechanically from massage, dry needling, stretching, or manual therapy? A systematic comparison of mechanical interventions is presented in **Table 1**.

**Table 1:** Comparison of mechanical interventions.

Feature	Acupuncture	Massage	Dry needling	Stretching
Scale	Micro-scale (0.2–0.3 mm needle diameter)	Macro-scale (hand/tool spanning centimeters)	Micro-scale (0.2–0.3 mm needle diameter)	Regional/whole-limb scale
Directionality	Three-dimensional (depth + rotation + lift-thrust); perpendicular to skin surface	Primarily tangential shear forces parallel to skin surface	Three-dimensional; perpendicular to skin	Longitudinal tensile along muscle-tendon axis
Depth of engagement	Deep fascia, subcutaneous tissue, muscle layers	Superficial to moderate depth	Muscle tissue, trigger points	Whole muscle-tendon unit across joints
Duration	Sustained 15–30 minutes with needle retention	Minutes of cyclic application	Brief seconds of rapid manipulation	Seconds to minutes of sustained hold
Manipulation type	Slow rotation, lifting-thrusting, intermittent stimulation; fascial winding around needle shaft	Compression, kneading, gliding, friction	Rapid pistoning; local muscle twitch response	Static or dynamic sustained hold; global lengthening of connective tissue
Force magnitude	Low sustained forces with intermittent high-strain events	Moderate cyclic compressive forces	Low-moderate brief forces	Moderate-high sustained tensile forces
Guarding response	Minimal (micro-scale insertion bypasses surface guarding)	Variable (may trigger protective muscle contraction)	Variable (twitch response is intentional)	Variable (stretch reflex may limit range)
Primary tissue target	Fascia, subcutaneous connective tissue	Skin, superficial fascia, muscle belly	Muscle trigger points, motor end plates	Muscle-tendon junction, joint capsule
Temporal effect profile	Cumulative over sessions (days–weeks)	Immediate relaxation, short-term effects	Immediate twitch, short-term relief	Immediate ROM increase, variable retention

### Scale of perturbation:

- Acupuncture: Micro-scale (needle diameter 0.2-0.3 mm), creating highly localized strain fields with minimal skin surface disruption (Leung et al., 2008; Ahn and Grodzinsky, 2009)
- Massage: Macro-scale (hand/tool compression spanning centimeters), creating diffuse pressure gradients (Weerapong et al., 2005; Field, 2014)
- Stretching: Whole-limb or regional, applying tensile loads across joints and muscle-tendon units (Magnusson et al., 2010; Page, 2012)

### Directionality:

- Acupuncture: Three-dimensional perturbation (insertion depth, rotation, lifting-thrusting), engaging deep fascial layers perpendicular to skin surface (Napadow et al., 2007; Zhou et al., 2011)
- Massage: Primarily tangential shear forces parallel to skin surface (Eagan et al., 2007; Berrueta et al., 2016)
- Dry needling: Similar needle geometry but different manipulation techniques and conceptual targeting (Cagnie et al., 2013; Dommerholt et al., 2019)

### Loading dynamics:

- Acupuncture: Sustained low-magnitude forces (needle retention 15–30 minutes) with intermittent manual manipulation creating transient high-strain events (Kong et al., 2007; Benham and Johnson, 2009)
- Massage: Cyclic compressive and shear loading over minutes (Goats, 1994; Sefton et al., 2012)
- Stretching: Sustained tensile loading maintained for seconds to minutes (Weppler and Magnusson, 2010; Konrad and Tilp, 2014)

**Figure 2:**
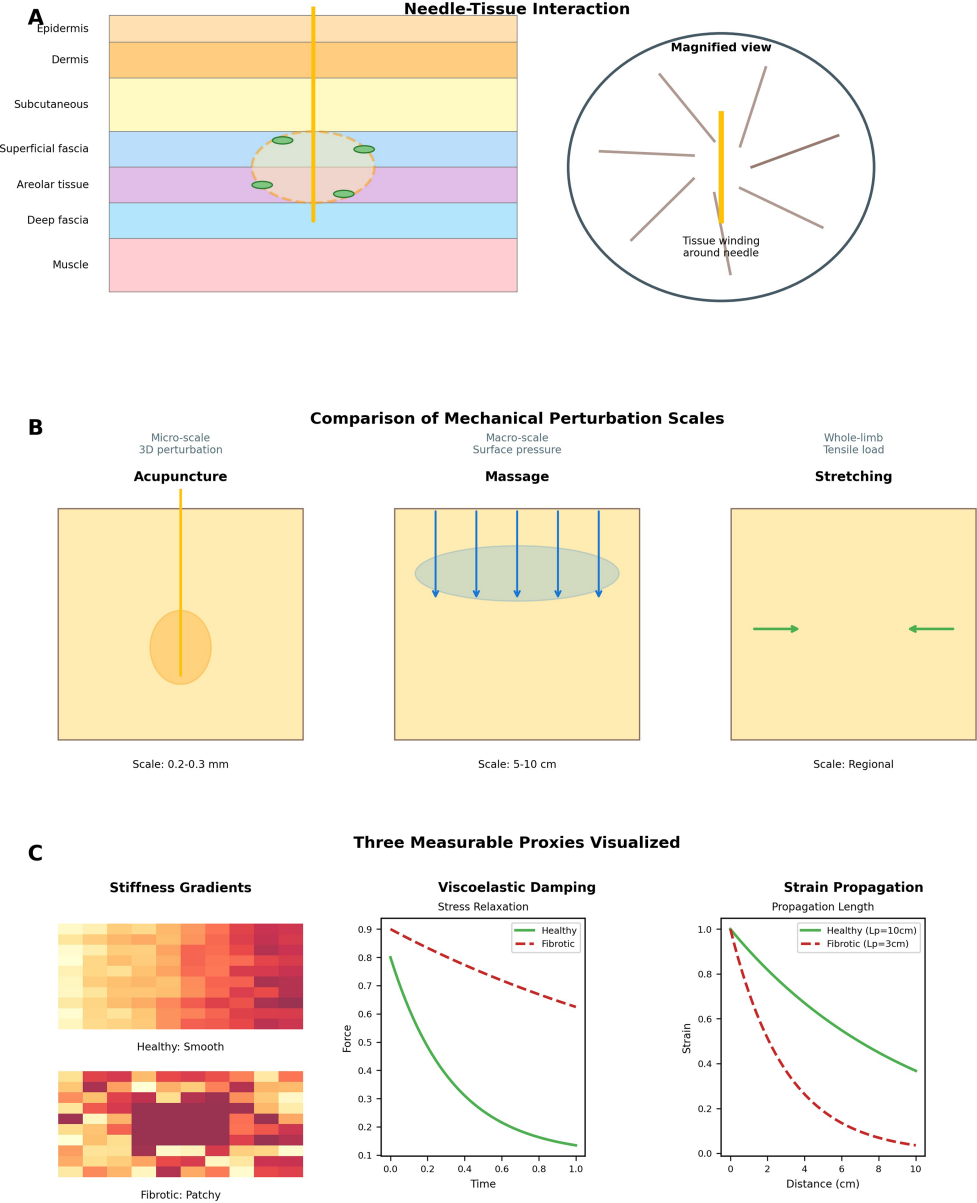
Acupuncture mechanical engagement and measurement proxies. **(A)** *Needle-Tissue Interaction.* Cross-sectional anatomical view showing acupuncture needle penetrating tissue layers. From surface: epidermis, dermis, subcutaneous adipose tissue with fascial septa, superficial fascia (dense connective tissue layer), loose areolar tissue, deep fascia, and underlying muscle. Magnified inset (circle) shows tissue winding mechanism: needle rotation causes collagen fibers and fascial strands to wrap around needle shaft, creating a zone of increased mechanical coupling extending several millimeters from needle. Fibroblasts within this zone (shown as elongated cells) experience cytoskeletal deformation and activate mechanotransduction pathways. **(B)** *Comparison of Mechanical Perturbation Scales.* Three panels showing strain field geometry for different interventions. Left (Acupuncture): Micro-scale perturbation with 0.2-0.3 mm diameter needle creating highly localized, three-dimensional strain field extending into deep fascial layers perpendicular to skin surface. Penetration depth 10–30 mm. Middle (Massage): Macro-scale perturbation with broad surface contact (5–10 cm span) creating diffuse pressure gradient affecting primarily superficial layers with tangential shear forces. Right (Stretching): Regional-scale perturbation with tensile forces distributed along entire muscle-tendon unit, creating longitudinal strain across joints. Each diagram shows characteristic strain field shape and affected tissue volume. **(C)** *Three Measurable Proxies Visualized.* Left (Stiffness Gradients): Elastography colormap of tissue region showing spatial heterogeneity. Healthy tissue (top) displays smooth gradients from higher stiffness (yellow/red, 15–25 kPa) to lower stiffness (blue/green, 5–10 kPa) with gradual transitions. Fibrotic tissue (bottom) shows abrupt boundaries, loss of gradient smoothness, and overall elevated values (20–40 kPa). Scale bar indicates kPa values. Middle (Viscoelastic Damping): Stress relaxation curves plotting normalized force versus time after step strain application. Healthy/plastic tissue (solid line) shows rapid initial decay and low residual force, indicating high damping (high tan δ). Fibrotic tissue (dashed line) shows slow decay and high residual force, indicating low damping (low tan δ). Equations show characteristic relaxation time constants (τ). Right (Strain Propagation): Schematic showing strain amplitude versus distance from loading point. Healthy tissue (solid line) shows gradual decay with long propagation length (Lp = 8–12 cm). Fibrotic tissue (dashed line) shows rapid decay with short propagation length (Lp = 2–4 cm). Propagation length defined as distance to 50% amplitude reduction.

Novel aspect of acupuncture: The combination of micro-scale access to deep fascial layers, three-dimensional perturbation, and sustained presence with intermittent manipulation creates a unique mechanical signature. Taken together, acupuncture occupies a distinct mechanical niche characterized by high spatial precision, low force magnitude, and deep fascial coupling—parameters not simultaneously achieved by other commonly used mechanical interventions (**Figure 2**).

### 4.3 Fibroblast mechanotransduction as primary mediator

Fibroblasts function as primary mechanosensors, translating mechanical cues into structural remodeling via:

- Integrin-mediated signaling: Mechanical forces transmitted through integrins activate focal adhesion kinase (FAK), Rho/ROCK pathways (Riento and Ridley, 2003; Schlaepfer et al., 2004; Parsons et al., 2010)
- YAP/TAZ mechanotransduction: Matrix stiffness directly regulates YAP/TAZ nuclear localization, controlling myofibroblast differentiation (Dupont et al., 2011; Liu et al., 2015; Haak et al., 2019)
- Cytoskeletal tension: Mechanical loading alters actin polymerization, stress fiber formation, and cellular contractility (Jaalouk and Lammerding, 2009; Burridge and Guilluy, 2016)

Empirical evidence for acupuncture-induced mechanotransduction: Langevin and colleagues demonstrated that acupuncture needle manipulation induces fibroblast cytoskeletal remodeling with increased cell spreading, enhanced motility, and changes in collagen fiber organization (Langevin et al., 2001b; Langevin et al., 2005; Langevin et al., 2007; Langevin et al., 2009; Langevin et al., 2010; Langevin et al., 2011b).

Conceptual hypothesis: Repeated mechanical perturbation through acupuncture may reduce pathological tissue stiffness, modify collagen alignment, and enhance viscoelastic damping capacity.

Temporal prediction: These processes occur over days to weeks, consistent with cumulative clinical effects reported following repeated acupuncture sessions (MacPherson et al., 2017; Vickers et al., 2018). However, whether these local cellular responses scale to tissue-level mechanical reorganization and system-wide functional effects remains unresolved and represents a critical gap in current evidence. A key unresolved issue is whether such local cellular responses exhibit sufficient spatial coherence to influence macroscopic strain distribution, rather than remaining confined to microstructural domains.

## 5 Measurable proxies for tensegrity and tissue plasticity

To enable empirical testing, the framework defines three primary biomechanical proxies:

### 5.1 Stiffness gradients

Definition: Spatial variation in tissue mechanical properties across anatomically relevant distances.

### Measurement methods:

- Ultrasound shear wave elastography (Gennisson et al., 2013; Barr et al., 2015)
- Myotonometry (Feng et al., 2018; Kelly et al., 2018)
- Indentation testing (Iivarinen et al., 2011; McKee et al., 2011)

Testable prediction: Acupuncture treatment should homogenize stiffness gradients toward physiological distribution.

### 5.2 Viscoelastic damping properties

Definition: Capacity of tissue to dissipate mechanical energy through time-dependent deformation and recovery.

### Measurement methods:

- Stress relaxation testing (Pinto and Fung, 1973; Suki et al., 2005)
- Creep testing (Thornton et al., 1997; Troyer and Puttlitz, 2011)
- Dynamic mechanical analysis measuring tan(δ) (Shen et al., 2011; Chaudhuri et al., 2020)

Testable prediction: Successful intervention should increase viscoelastic damping (higher tan δ, faster stress relaxation, reduced creep).

### 5.3 Strain-propagation length

Definition: Spatial extent over which mechanical deformation spreads from a point of loading.

### Measurement methods:

- Imaging-based deformation analysis (D’hooge et al., 2000; Wang and Larin, 2015)
- Multi-site mechanical impedance (Gefen et al., 2002; Vain et al., 2015)
- Computational modeling (Kruse et al., 2000; Sinkus et al., 2005)

Testable prediction: Treatment should increase strain-propagation length, indicating improved global force redistribution.

### 5.4 Integration of multiple proxies

No single proxy fully captures tissue plasticity. Combined assessment enables identification of dominant mechanical constraints and phenotype-guided treatment strategies. A critical implication is that discordant changes between proxies—for example, normalization of stiffness without corresponding increases in strain-propagation length—would challenge the validity of a tensegrity-based interpretation. Definitions, measurement methods, and predicted changes for each proxy are summarised in **Table 2**.

**Table 2:** Measurable proxies for tissue plasticity assessment.

Proxy	Definition	Measurement tools	Technical parameters	Predicted change with successful treatment	Clinical interpretation
Stiffness Gradients	Spatial heterogeneity in tissue elastic modulus across anatomically relevant distances	Ultrasound shear wave elastography; Myotonometry (MyotonPRO); Indentation testing; Atomic force microscopy	Resolution: 1–2 mm (ultrasound), 10 µm (AFM); Units: kPa or N/m	Homogenization toward physiological distribution; Reduced peak stiffness in hypertonic regions; Increased stiffness in hypotonic regions	Loss of heterogeneity indicates impaired regional specialization; Normalization suggests restored tensegrity function
Viscoelastic Damping (tan δ)	Capacity to dissipate mechanical energy through time-dependent deformation and recovery; Phase lag between stress and strain	Stress relaxation testing; Creep testing; Dynamic mechanical analysis (DMA); Shear wave dispersion ultrasound	tan(δ) = Loss modulus / Storage modulus; Relaxation time constant (τ); Creep compliance J(t)	Increased tan(δ); Faster stress relaxation (shorter τ); Reduced creep (lower J at steady state)	Low damping indicates elastic/fibrotic behavior with poor energy absorption; Restored damping indicates improved mechanical resilience
Strain-Propagation Length	Spatial extent over which mechanical deformation spreads from a point of loading	Ultrasound speckle tracking; Digital image correlation; Optical coherence tomography; Multi-site impedance measurement; Finite element modeling	Propagation length in mm or cm; Decay constant of strain amplitude with distance	Increased propagation length; More uniform strain distribution; Reduced mechanical compartmentalization	Short propagation indicates fibrotic compartmentalization with local overload; Long propagation indicates effective tensegrity network function

## 6 Relation to existing literature and novel contributions

### 6.1 Fibroblast and mechanotransduction literature

Established knowledge: Matrix stiffness regulates fibroblast phenotype; integrin-FAK-Rho/ROCK pathways transduce mechanical signals; YAP/TAZ are key mechanosensitive regulators (Riento and Ridley, 2003; Schlaepfer et al., 2004; Engler et al., 2006; Jaalouk and Lammerding, 2009; Parsons et al., 2010; Dupont et al., 2011; Yang et al., 2014; Liu et al., 2015; Burridge and Guilluy, 2016; Haak et al., 2019).

Novel contribution: Integrates these molecular mechanisms into a systems-level tensegrity architecture where cellular mechanotransduction is constrained by tissue-level mechanical boundary conditions.

### 6.2 Fascia research

Established knowledge: Fascia forms continuous networks with active contractility and mechanoreceptors (Yahia et al., 1992; Stecco et al., 2009; Stecco et al., 2011; Tesarz et al., 2011; Hinz et al., 2012; Adstrum et al., 2017; Benias et al., 2018; Schleip et al., 2019).

Novel contribution: Positions fascia as the primary substrate for tensegrity-based force redistribution with testable biomechanical hypotheses.

### 6.3 Acupuncture mechanism research

Established knowledge: Acupuncture mechanically engages tissue, induces fibroblast remodeling, and activates neural/endocrine pathways (Langevin et al., 2001a; Langevin et al., 2001b; Langevin et al., 2002; Han, 2004; Langevin et al., 2004; Langevin et al., 2005; Langevin et al., 2006; Langevin et al., 2007; Dorsher and Fleckenstein, 2008; Zhao, 2008; Langevin et al., 2009; Langevin et al., 2010; Langevin et al., 2011a; Langevin et al., 2011b; Huang et al., 2012; Langevin et al., 2013; MacPherson et al., 2017; Vickers et al., 2018).

Novel contribution: Explicitly positions mechanical mechanisms as primary with quantifiable proxies, specific predictions about responder phenotypes, and temporal dynamics.

### 6.4 Summary of novel contributions

1. Architectural integration connecting acupuncture to tensegrity and fibrosis–plasticity continuum
2. Mechanistic specificity defining how acupuncture differs from other interventions
3. Testable predictions with measurable proxies
4. Systems-level perspective explaining non-local effects
5. Clinical phenotyping for treatment prediction

### 6.5 Critical engagement with contemporary literature

#### Challenges from the fascial and connective tissue literature

A significant challenge to the present framework comes from studies demonstrating that force transmission through fascial layers is strongly attenuated by tissue sliding at fascial interfaces. Huijing and colleagues have shown that myofascial force transmission between muscles can be substantial in passive conditions but is modulated by the degree of inter-fascial sliding, with pathological adhesion or excessive lubrication each disrupting normal transmission (Huijing and Baan, 2003). This implies that tensegrity-like non-local effects depend critically on the integrity of interfascial sliding mechanics — a dimension the present framework acknowledges only implicitly. Future operationalizations should incorporate fascial gliding capacity as a measurable variable alongside stiffness gradients.

#### Challenges from acupuncture sham-controlled trials

The most substantive challenge to mechanism-specific claims for acupuncture comes from the systematic finding that sham acupuncture (superficial needling, non-acupoint insertion, or non-penetrating Streitberger needles) frequently produces effects comparable to verum acupuncture in randomized controlled trials (Madsen et al., 2009). This represents the strongest empirical challenge to any mechanism-specific interpretation of acupuncture and must be explicitly addressed rather than reinterpreted within the model’s assumptions. This interpretation further implies that differences between verum and sham interventions should emerge only when perturbation depth, spatial precision, or coupling to deep fascial layers differ sufficiently to alter strain propagation.

#### Alignment with and divergence from the Langevin connective tissue program

The present framework is most directly aligned with the research program of Langevin and colleagues, who have provided the strongest empirical foundation for connective tissue-mediated effects of acupuncture (Langevin et al., 2001a; Langevin et al., 2001b; Langevin et al., 2002; Langevin et al., 2004; Langevin et al., 2005; Langevin et al., 2006; Langevin et al., 2007; Langevin et al., 2009; Langevin et al., 2010; Langevin et al., 2011a; Langevin et al., 2011b; Langevin et al., 2013). A key divergence, however, is interpretative: Langevin’s program has primarily characterized local cellular responses to needle manipulation, whereas the present framework extends this to system-level tensegrity architecture and non-local force redistribution. This extension requires additional justification. The assumption that local fibroblast remodeling propagates to system-level architectural change is plausible given the continuity of the fascial network but has not been directly demonstrated across spatial scales in the context of acupuncture.

#### Convergences with the mechanobiology of ageing and fibrosis

The framework’s core claim — that tissue architectural state determines recovery capacity — converges strongly with recent mechanobiological evidence that matrix stiffness acts as a primary driver of fibroblast phenotype independently of biochemical environment (Engler et al., 2006; Dupont et al., 2011; Yang et al., 2014; Liu et al., 2015; Haak et al., 2019). The YAP/TAZ pathway in particular provides a molecular mechanism through which mechanical perturbation could shift fibroblast behavior from pro-fibrotic to pro-remodeling phenotypes. This convergence strengthens the biological plausibility of the framework and suggests that future experimental designs should incorporate YAP/TAZ nuclear localization as a mechanistic readout alongside the biomechanical proxies proposed.

## 7 Boundary conditions: when do neural/neuroendocrine mechanisms dominate?

### 7.1 Temporal boundaries

### Neural/neuroendocrine mechanisms dominate in:

- Immediate responses (seconds to minutes): autonomic reflexes, endorphin release (Han, 2004; Zhao, 2008; Langevin et al., 2011c)

Acute stress states: HPA axis activation, cytokine release (McEwen, 2007; Chrousos, 2009).

### Mechanical mechanisms dominate in:

- Delayed responses (hours to weeks): fibroblast remodeling, collagen reorganization (Langevin et al., 2005; Kjaer et al., 2009; Balestrini et al., 2012)
- Cumulative effects: progressive improvement over multiple sessions (MacPherson et al., 2017; Vickers et al., 2018)

### 7.2 Clinical phenotype boundaries

### Neural/neuroendocrine mechanisms dominate in:

- Functional disorders without structural constraints (Mayer et al., 2015; Drossman and Hasler, 2016)
- Acute nociceptive states with neural sensitization (Latremoliere and Woolf, 2009; Hodges and Tucker, 2011)
- Psychophysiological conditions (Sarno, 1998; Henningsen et al., 2007)

### Mechanical mechanisms dominate in:

- Chronic structural conditions with measurable tissue changes (Wells et al., 2003; Kass et al., 2004; Provenzano and Vanderby, 2006; Borlaug and Paulus, 2011; King et al., 2014; Rockey et al., 2015; Scarcelli et al., 2015; Henderson et al., 2020)
- Age-related stiffness, post-injury scarring, fibrotic organ disease

### 7.3 Individual variability

Neuroendocrine dominance: High stress reactivity (Porges, 2001; Thayer et al., 2012), central sensitization (Curatolo et al., 2006; Woolf, 2011), psychological factors (Sullivan et al., 2001; Kaptchuk et al., 2008).

Mechanical dominance: Measurable tissue stiffness, stable autonomic function, visible functional constraints.

### 7.4 Interaction and hierarchy

The framework proposes hierarchical interaction:

- Primary layer: Tissue-level mechanical architecture constrains or enables function
- Secondary layer: Neuroendocrine mechanisms modulate speed, variability, and subjective experience

**Figure 3:**
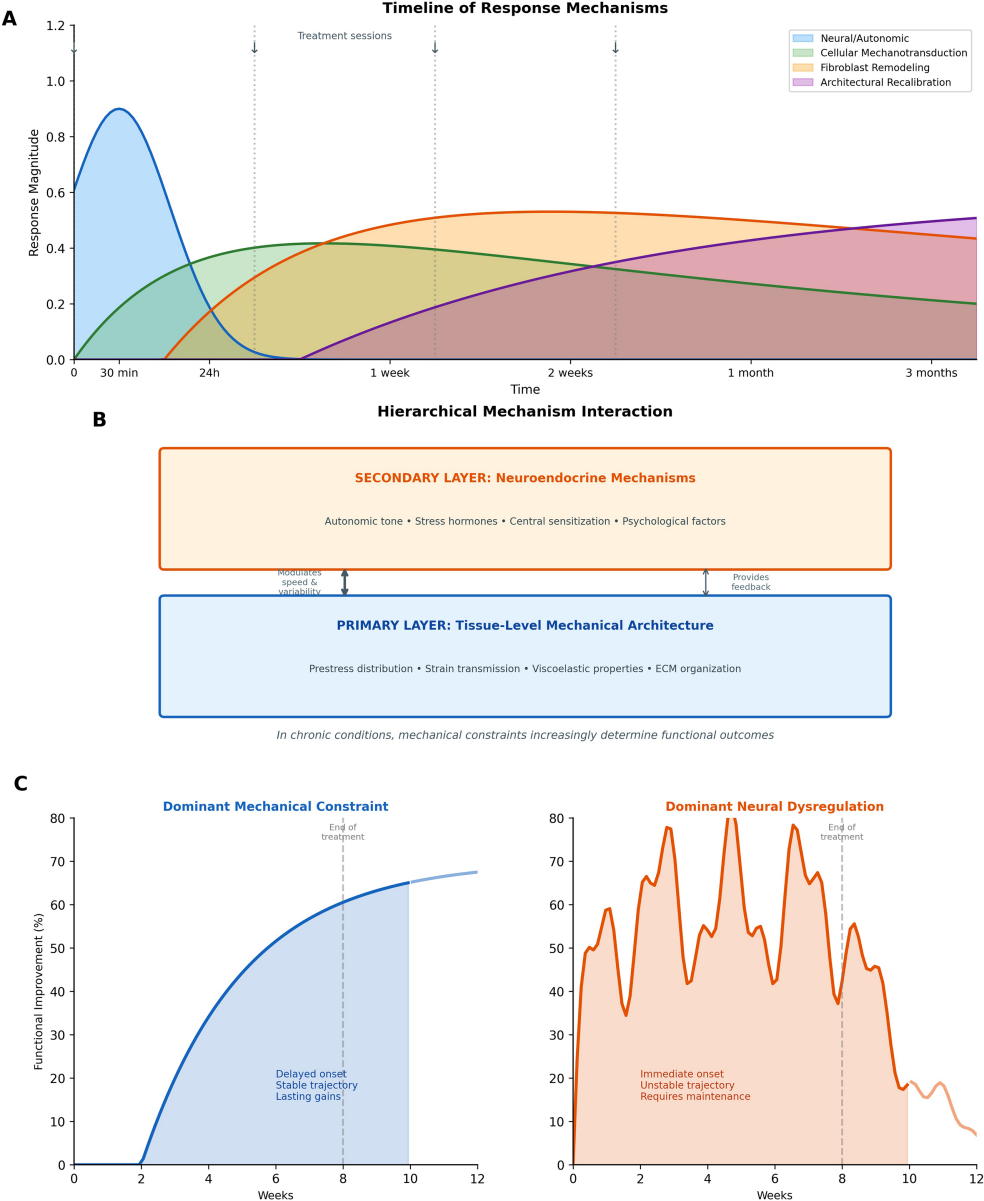
Temporal dynamics and mechanism hierarchy. **(A)** *Timeline of Response Mechanisms.* Horizontal timeline from immediate (seconds) to sustained (months) showing four overlapping phases of acupuncture response. Immediate (0–30 min): Neural reflex activation (autonomic modulation, endorphin release, acute analgesia) – shown in blue, peaks rapidly, decays within hours. Early (30 min – 24 h): Cellular mechanotransduction activation (integrin signaling, YAP/TAZ translocation, cytokine modulation) – shown in green, rises over hours, persists 1–2 days. Delayed (1–14 days): Fibroblast remodeling responses (cell spreading, collagen reorganization, ECM turnover) – shown in orange, rises over days, peaks at 1–2 weeks. Sustained (weeks-months): Architectural recalibration (restored prestress distribution, improved strain propagation, normalized viscoelasticity) – shown in red, gradual rise, plateau with sustained treatment. Cumulative treatment sessions indicated by arrows along timeline. **(B)** *Hierarchical Mechanism Interaction.* Layered diagram showing relationship between mechanical and neuroendocrine mechanisms. Bottom (Primary Layer): Tissue-level mechanical architecture including prestress distribution, strain transmission capacity, viscoelastic properties, and ECM organization. This layer constrains or enables function and determines recovery potential. Forms the architectural substrate. Top (Secondary Layer): Neuroendocrine mechanisms including autonomic tone (sympathetic/parasympathetic balance), stress hormones (cortisol, catecholamines), central sensitization state, and psychological factors. This layer modulates speed, variability, and subjective experience of changes occurring at the mechanical level. Bidirectional arrows indicate interaction: mechanical changes alter proprioceptive input affecting neural state; neural state affects muscle tone and vascular supply affecting tissue mechanics. However, arrow thickness indicates that in chronic conditions, mechanical constraints increasingly determine functional outcomes. **(C)** *Predicted Response Patterns by Phenotype.* Two contrasting response trajectories over 8-week treatment course (x-axis: weeks 0-8; y-axis: functional improvement %). Left graph (Dominant Mechanical Constraint): Patient with high tissue stiffness, normal autonomic function. Week 0-2: Minimal change despite treatment (dotted line near baseline). Week 2-4: Gradual improvement begins. Week 4-8: Continued incremental gains reaching 60-70% improvement. Post-treatment (weeks 8-12, shown faded): Sustained benefit without continued treatment. Characteristics: Delayed onset, stable trajectory, lasting gains. Right graph (Dominant Neural Dysregulation): Patient with normal tissue mechanics, high autonomic reactivity. Week 0-1: Rapid initial improvement to 50-60% (solid line rises steeply). Week 1-4: Fluctuating course with variable responses between sessions (oscillating line). Week 4-8: Average improvement may be similar but unstable. Post-treatment: Rapid return toward baseline without continued treatment. Characteristics: Immediate onset, unstable trajectory, requires maintenance. Middle graph (Mixed Presentation): Biphasic response showing initial neural component (rapid early rise) followed by mechanical component (continued gradual improvement), with intermediate stability.

Testable hypothesis: Patients with high stiffness but normal autonomic function should show delayed but sustained benefit. Patients with normal mechanics but high autonomic reactivity should show immediate but unstable benefit (**Figure 3**). In complex clinical states, neural and mechanical mechanisms may generate opposing or temporally misaligned effects, potentially explaining unstable or paradoxical treatment responses.

## 8 Clinical implications and phenotype-guided strategies

### 8.1 Matching intervention to tissue state

Patients with dominant architectural impairment (age-related stiffness, chronic fibrotic changes). Predicted response patterns by clinical phenotype are presented in **Table 3**.

**Table 3:** Predicted response patterns by clinical phenotype.

Clinical phenotype	Dominant mechanism	Pre-treatment characteristics	Expected response timeline	Response pattern	Recommended intervention duration	Prognosis
High tissue stiffness + Normal autonomic function	Mechanical (architectural)	Elevated elastography values; Restricted ROM; Normal HRV; Low psychological distress	Days to weeks (delayed onset)	Gradual, incremental improvement; Stable gains between sessions; Sustained after treatment completion	8–12 sessions over 4–8 weeks, then tapering	Good if plasticity can be restored; May plateau at partial improvement
Normal tissue mechanics + High autonomic reactivity	Neuroendocrine (neural)	Normal elastography; Full ROM; Low HRV or high variability; High psychological distress	Minutes to hours (immediate)	Rapid initial improvement; Fluctuating between sessions; Unstable; Relapse when treatment stops	Ongoing intermittent PRN; Address underlying stress factors	Variable; Requires addressing neural/psychological factors
Mixed presentation	Both mechanical and neural	Moderate stiffness; Moderate ROM restriction; Variable HRV; Moderate psychological factors	Variable (biphasic)	Initial neural response (hours); Later mechanical response (weeks); Combined trajectory	6–8 weeks regular treatment; Then variable maintenance	Intermediate; Benefits from multimodal approach
Acute functional disorder	Neuroendocrine (neural)	Normal tissue mechanics; Acute onset symptoms; High stress context; No structural changes	Immediate (minutes)	Rapid, potentially complete resolution; Single or few sessions effective	1–4 sessions	Excellent if addressed promptly
Chronic structural/fibrotic	Mechanical (architectural)	High stiffness on elastography; Imaging-confirmed fibrosis; Chronic duration (years); Normal autonomic function	Weeks to months (very delayed)	Slow, partial improvement; May plateau below normal; Some irreversibility	12–16+ sessions over 3–4 months; Long-term maintenance	Guarded; Structural changes may limit full recovery
Post-injury scarring	Mechanical (architectural)	Localized high stiffness; History of trauma/surgery; Palpable tissue texture changes	Weeks (delayed)	Gradual improvement; May be limited by scar maturity	8–12 sessions; Consider combination with other physical therapies	Moderate; Depends on scar age and extent
Age-related stiffness	Mechanical (architectural)	Diffuse increased stiffness; Progressive onset; Multiple body regions; No acute trigger	Weeks to months	Slow improvement; May require ongoing treatment; Lifestyle factors important	Long-term maintenance approach; 4–8 sessions then monthly	Fair; Slowing progression may be realistic goal

- Minimal immediate effect
- Gradual improvement over 4-8+ sessions
- Requires longitudinal intervention
- Benefit sustained after treatment completion

Patients with dominant neuro-functional dysregulation (acute stress, functional syndromes):

- Rapid initial improvement
- Potentially unstable responses
- May require ongoing intermittent treatment
- Risk of relapse without addressing underlying factors

A direct prediction of this framework is that patients with dominant mechanical constraints should not exhibit rapid placebo-like responses, a hypothesis that can be tested in stratified clinical studies. Failure to observe such stratified response patterns would directly challenge the proposed hierarchy between mechanical and neuroendocrine mechanisms.

### 8.2 Integration with conventional therapies

The framework suggests acupuncture as complementary to conventional approaches:

- Molecular therapies address biochemical drivers
- Mechanical therapies address architectural constraints
- Neural therapies address amplification factors

## 9 Limitations and future directions

### 9.1 Framework limitations

Key limitations:

1. Limited direct evidence for the integrated framework
2. Simplified representation of complex biological tissues
3. Measurement challenges with current technologies
4. Individual heterogeneity not yet predictable
5. The central assumption that localized mechanical perturbations can scale to system-level tensegrity effects remains unproven and may ultimately prove incorrect, representing a key point of potential falsification

### 9.2 Testable predictions for future research

1. Stiffness normalization measurable via elastography
2. Viscoelastic recovery (increased tan δ, faster relaxation)
3. Strain propagation predicting treatment response
4. Temporal dynamics distinguishing mechanical from molecular changes
5. Phenotype stratification validating mechanical assessment

### 9.3 Proposed experimental designs

Study 1: Acute mechanical effects with pre/post elastography

Study 2: Longitudinal remodeling over 8 weeks with weekly assessments

Study 3: Mechanism dissection comparing acupuncture vs. sham vs. manual pressure

### 9.4 Explicit model assumptions

The framework rests on a set of structural assumptions that delimit its scope and require acknowledgement as conditions for the model’s internal coherence. First, it assumes that tensegrity principles scale across biological levels — from cytoskeletal filaments to fascial networks — in a manner that preserves the core mechanical properties of prestress and non-local force redistribution (Ingber, 2003a; Ingber, 2003b; Ingber, 2006). While hierarchical tensegrity has strong theoretical and experimental support at molecular and cellular levels (Ingber, 2006; Geiger et al., 2009; Hynes, 2009; Wang et al., 2009), its extension to whole-body fascial networks remains a productive hypothesis rather than an established fact.

Second, the model assumes that fibrosis and plasticity are not dichotomous states but poles of a continuous mechanical spectrum. This continuum assumption implies that intermediate architectural states exist and are clinically relevant, and that interventions can shift tissue position along this axis without requiring complete structural reversal. The assumption is supported by the graded nature of fibroblast mechanosensing and ECM remodeling (Lutolf and Hubbell, 2005; Engler et al., 2006; Humphrey et al., 2014; Yang et al., 2014) but has not been validated as a continuous measurable variable in clinical populations.

Third, the framework assumes that acupuncture needle manipulation generates mechanically distinct perturbations that are qualitatively different from sham procedures, dry needling, or massage, owing to its unique combination of micro-scale access, three-dimensional strain vectors, and sustained presence with intermittent stimulation. This assumption is supported by empirical measurements of tissue winding (Langevin et al., 2004; Langevin et al., 2011a) and fibroblast cytoskeletal remodeling (Langevin et al., 2001a; Langevin et al., 2001b; Langevin et al., 2005; Langevin et al., 2006; Langevin et al., 2007; Langevin et al., 2009; Langevin et al., 2010; Langevin et al., 2011b), but the functional equivalence or non-equivalence of different needle techniques has not been resolved.

Fourth, neuroendocrine pathways are assigned a secondary modulatory role rather than primary causal status. This hierarchical assumption requires empirical dissection and may not hold across all clinical phenotypes, particularly in conditions where central sensitization or autonomic dysregulation precedes structural change. Together, these assumptions define a testable but non-trivial model that is vulnerable to empirical refutation if cross-scale mechanical coupling cannot be demonstrated.

### 9.5 Interpretative limits

The framework should not be interpreted as a complete mechanistic explanation of acupuncture’s clinical effects. Its interpretative scope is bounded in several important ways. The model addresses how acupuncture may influence tissue architecture through tensegrity mechanics; it does not address whether specific clinical outcomes are attributable to this mechanism in any given patient or condition. The measurable proxies proposed (stiffness gradients, viscoelastic damping, strain-propagation length) are necessary but not sufficient evidence: changes in these parameters would support but not confirm the tensegrity interpretation, since identical biomechanical changes could in principle result from neuromodulatory or vascular mechanisms.

The framework also does not resolve the question of acupuncture point specificity. Tensegrity-mediated force redistribution is topology-dependent, meaning that different insertion sites will engage different mechanical pathways; however, whether classical acupoint locations correspond to mechanically privileged nodes in the fascial network is an open empirical question that the present model cannot adjudicate. Similarly, the model does not address dosing parameters (needle gauge, insertion depth, retention duration, stimulation frequency) beyond qualitative characterization; dose–response relationships at the tissue level remain to be determined.

## 10 Conclusion

Fibrosis reflects a collapse of tissue plasticity within a biomechanical continuum. Tensegrity theory provides a coherent framework to understand this collapse and its potential reversibility.

By positioning acupuncture as a localized mechanical perturbation within a prestressed connective tissue network, this framework:

- Avoids metaphysical explanations while preserving clinical observations
- Generates testable predictions using measurable biomechanical proxies
- Explains non-local effects through architectural principles
- Distinguishes mechanical from neural mechanisms
- Provides basis for phenotype-guided treatment strategies

The framework’s value lies in integrating a reproducible mechanical intervention into contemporary biomechanics and systems physiology. Whether acupuncture proves effective for modulating tissue plasticity remains an empirical question. If validated, this framework would reposition acupuncture from a debated therapeutic modality to a quantitative probe of human tissue architecture.

## Data Availability

The original contributions presented in the study are included in the article/supplementary material. Further inquiries can be directed to the corresponding author.
